# Evaluation of Brain Function Recovery After Traumatic Brain Injury Treatment in a Porcine Model by Cross-Group Temporal–Spatial Correlation Analysis

**DOI:** 10.1089/neur.2023.0059

**Published:** 2024-07-01

**Authors:** Wenwu Sun, William Reeves, Madison M. Fagan, Christina B. Welch, Kelly M. Scheulin, Sydney E. Sneed, Todd R. Callaway, Kylee J. Duberstein, Franklin D. West, Qun Zhao

**Affiliations:** ^1^ Department of Physics and Astronomy, University of Georgia, Athens, Georgia, USA.; ^2^ University of Georgia, Regenerative Bioscience Center, Athens, Georgia, USA.; ^3^ Bio-Imaging Research Center, University of Georgia, Athens, Georgia, USA.; ^4^ Neuroscience Program, Biomedical and Health Sciences Institute, University of Georgia, Athens, Georgia, USA.; ^5^ Department of Animal and Diary Science, University of Georgia, Athens, Georgia, USA.

**Keywords:** functional MRI, independent component analysis, porcine model, temporal–spatial analysis, traumatic brain injury

## Abstract

Traumatic brain injury (TBI), a significant global health issue, is affecting ∼69 million annually. To better understand TBI’s impact on brain function and assess the efficacy of treatments, this study uses a novel temporal–spatial cross-group approach with a porcine model, integrating resting-state functional magnetic resonance imaging (rs-fMRI) for temporal and arterial spin labeling for spatial information. Our research used 18 four-week-old pigs divided into three groups: TBI treated with saline (SLN, *n* = 6), TBI treated with fecal microbial transplant (FMT, *n* = 6), and a sham group (sham, *n* = 6) with only craniectomy surgery as the baseline. By applying machine learning techniques—specifically, independent component analysis and sparse dictionary learning—across seven identified resting-state networks, we assessed the temporal and spatial correlations indicative of treatment efficacy. Both temporal and spatial analyses revealed a consistent increase of correlation between the FMT and sham groups in the executive control and salience networks. Our results are further evidenced by a simulation study designed to mimic the progression of TBI severity through the introduction of variable Gaussian noise to an independent rs-fMRI dataset. The results demonstrate a decreasing temporal correlation between the sham and TBI groups with increasing injury severity, consistent with the experimental results. This study underscores the effectiveness of the methodology in evaluating post-TBI treatments such as the FMT. By presenting comprehensive experimental and simulated data, our research contributes significantly to the field and opens new paths for future investigations into TBI treatment evaluations.

## Introduction

Traumatic brain injury (TBI) is a significant global health issue, affecting ∼69 million people annually.^1^ As the leading cause of death and disability for individuals younger than 45 years,^2^ understanding TBI’s effects on brain function (e.g., connectivity) and evaluating the impact of various treatments are essential. Functional magnetic resonance imaging (fMRI) holds great potential for assessing alterations in brain functional connectivity post-TBI by tracking network recovery effects because of novel therapeutics.^3,4^

The pig model has become popular for TBI and other neurological disease studies (e.g., stroke)^5,6^ as a translational large animal model with similar size and neuroanatomy^6^ with human. Research using the pig TBI model has increased, as it is more likely to predict human outcomes and contribute to improved therapeutic devices and pharmacological treatment development. Our team recently demonstrated that pigs have homologous resting-state functional networks to human brains^7^ and identified functional connectivity disruptions because of TBI,^5^ further emphasizing the pig model’s importance for studying functional network changes.

Various methods have been applied to reveal functional connectivity, including model-free methods such as sparse dictionary learning (sDL)^5,7,8^ and independent component analysis (ICA),^9^ as well as seed-based methods^10^ and functional connectome-based cross-subject analysis.^11,12^ As pigs require anesthesia during MRI scanning to reduce motion artifacts, task-based fMRI meets many challenges. Existing resting-state fMRI (rs-fMRI) methods mostly use the spatial correlation between a known atlas and generated activation maps^5^ to evaluate recovery of brain functions after TBI, whereas temporal correlation (e.g., the FSLNets^9^) has been used for internetwork comparisons within a group. However, neither spatial nor temporal correlation analysis has used a common baseline (e.g., a sham group in our porcine TBI study) for evaluating brain damages or disruptions of functional connectivity.

To evaluate TBI recovery and treatment effects, we introduced a novel method for cross-group correlation analysis, drawing inspiration from FSLNets’ cross-group modeling and the broader framework of functional connectome studies, which has been proven important for individual fingerprinting.^13^ Our correlation studies assessed functional activity similarity between a TBI group and a sham group, where the sham group underwent only craniotomy surgery,^14^ and the sham group’s resting-state networks (RSNs) were considered to maintain normal functional connectivity. We extracted time series of RSNs from both TBI and sham groups’ raw fMRI data using sDL or ICA for functional analyses. Those time series were then fed to FSLNets to investigate correlations between these groups, where a higher correlation with the sham group over two time points suggested improved brain function recovery compared with lower or unchanged correlations. We compared these temporal correlation findings with cerebral blood flow (CBF) maps derived from arterial spin labeling (ASL) data. Consistent with previous studies that demonstrated a strong spatial correlation between CBF maps and functional connectivity patterns, our analysis revealed that both the CBF spatial results and the temporal correlation trends exhibited similar patterns, reinforcing the validity of our findings in tracking recovery after TBI.

To better understand the TBI evaluation results, our study uses a simulation that mimics the progression of TBI from mild to severe by adding varying levels of Gaussian noise to experimental data in a larger pig dataset. This step was crucial for testing the sensitivity of our methods in detecting the nuances of recovery, ensuring that our findings could offer significant insights into the dynamic processes of healing post-TBI. Our results reveal distinct differences across the spectrum of injury severity, further validated by comparison with real TBI data.

This study contributes two major advancements compared with our previous research. First, it uses a temporal–spatial correlation analysis strategy for functional connectivity. Second, it uncovers functional changes in TBI groups by comparing network behaviors with a common baseline (i.e., a sham group) at two post-TBI time points.

## Methods

### Animal preparation and MRI data acquisition

A total of 18 castrated 4-week-old male crossbreed piglets were used in this study. They were randomly assigned to one of three groups: sham craniectomy + saline (*n* = 6, referred to as sham group), TBI + saline (*n* = 6, referred to as SLN group), and TBI + fecal microbial transplant (*n* = 6, referred to as FMT group). FMT or saline was administered by oral gavage beginning 2h post injury and then every 24h for 7 days. The moderate-to-severe TBI was conducted using our previously published procedure.^15–17^ Briefly, all pigs were anesthetized and underwent a 20 mm craniectomy surgery at the left anterior junction of the coronal and sagittal sutures. Pigs were then secured in a controlled cortical impactor device, and a 15 mm impactor tip was positioned over the intact dura to induce injury in the sensorimotor network region^5^ with the following parameters: velocity of 4 m/s, depth of depression of 9 mm, and dwell time of 400 ms. All experimental procedures were approved by the Institutional Animal Use and Care Committee at the University of Georgia.

MRI was conducted on all pigs at two time points, day 1 (D1) and day 7 (D7) postsurgery. Pigs were anesthetized, and mild anesthesia was maintained via inhalation isoflurane for the duration of the scans. T1-weighted anatomical, rs-fMRI, and ASL data were collected using a GE 32-channel fixed-site Discovery MR750 3.0 Tesla magnet and an 8-channel knee coil. T1-weighted anatomical, rs-fMRI, and ASL data were acquired using the following sequences: (1) 3D fast spoiled gradient echo sequence (repetition time [TR] = 5.5 sec, echo time [TE] = 2.1 ms, flip angle [FA] = 9°, field of view [FOV] = 12.8 × 12.8 × 6.4 cm, slice thickness = 1 mm, a reconstruction matrix size of 256 × 256 × 112 [resulting in cubic voxels of 0.5 mm], axial slice plane, and an acquisition time of 10 min 57 sec); (2) gradient echo-planar imaging sequence (TR = 3 sec, TE = 30 ms, FA = 80°, FOV = 12.8 × 12.8 × 6.2 cm, a matrix size of 96 × 96 × 31, coronal slice plane, 305 total volumes for rs-fMRI, and an acquisition time of 15 min 15 sec); and (3) 3D pseudo-continuous ASL sequence (FOV = 12.8 cm x 12.8 cm, slice thickness 3 mm, and frequency/phase encoding of 512 and 8).

### Data preprocessing and RSN node selection

First, all rs-fMRI and ASL images were registered to a reference space using statistical parametric mapping (SPM12) in MATLAB 2021b. A whole-brain masking was performed for each subject to isolate the brain from the surrounding tissues, and the brain was subsequently registered to the T1-weighted images in the anatomical space.

In this study, two machine learning models, ICA (FSL MELODIC^9^) and sDL,^18^ were applied for temporal analysis, aiming to reveal the effect of recovery from TBI. We divided the data into two distinct datasets for machine learning analysis: the full dataset learning (FL), containing rs-MRI dataset of both TBI and sham groups (sham+SLN+FMT), and the sham dataset learning (SL), which includes only dataset of the sham group (sham). The FL dataset, featuring a broader subject set, was intended to capture a comprehensive representation of recovery process covering both TBI and craniectomy. In contrast, the SL dataset focused on establishing a baseline with standard features from the sham group (craniectomy) alone. Applying either ICA or sDL to either FL or SL datasets led to four experimental conditions, thereby facilitating a thorough assessment of result consistency across these varied scenarios.

For each experimental condition, ICA was used to identify 100 group-level independent components, and sDL was used to detect 300 atoms, with both independent components and atoms representing distinctive patterns of brain functions. These identified components and atoms were then projected back to coregistered, template pig brain’s T1-images for mapping of various brain functions, thresholded by a z-score of 1, smoothed, and correlated with seven predefined atlases of RSNs (as elaborated in Table 1). The component or atom exhibiting the highest Pearson correlation with any of the seven RSNs was chosen as its corresponding representative RSN node. Leveraging the FSL dual regression technique, we extracted the time series for each subject corresponding to the seven RSN nodes. These time series were then analyzed to construct functional connectivity matrices, offering insights into brain functional connectivity patterns.

### Cross-group temporal correlation analysis

As the focus of this study is the evaluation of post-TBI brain function recovery, a baseline is necessary for cross-group evaluations. As the sham group pigs received only the craniectomy surgery and saline, their RSNs are assumed to maintain normal functional activities, accompanied by the craniectomy effect at different time points (D1 and D7). This hypothesis enables us to use the sham group-based RSNs as the baseline for brain function recovery evaluation of TBI groups.

**FIG. 1.**
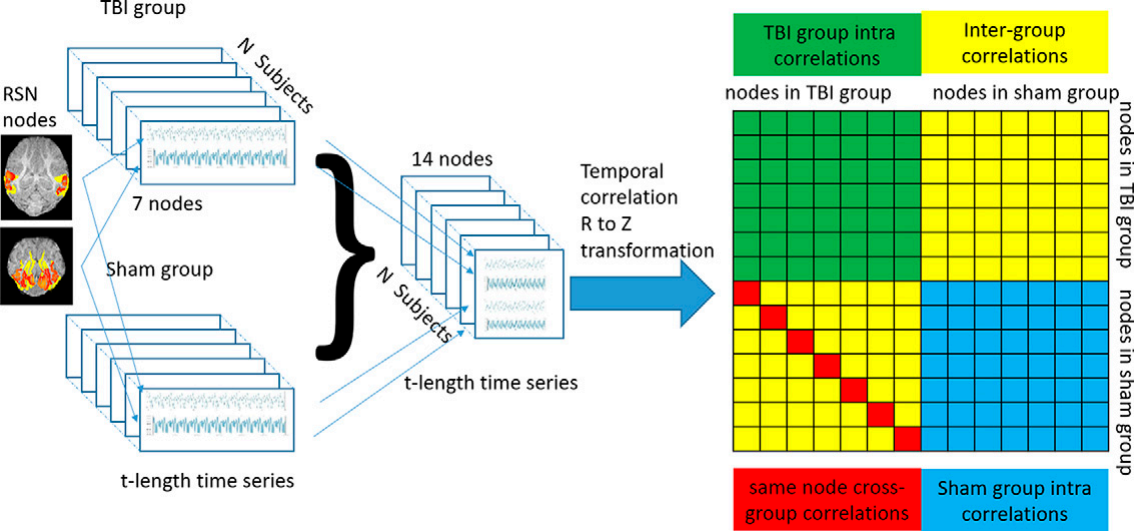
Flowchart depicting the cross-group temporal analysis methodology. Time series are rearranged to form pseudo-subjects, doubling the node number for FSLNets functions. In the FSLNets’ node-by-node matrix output, the red diagonal of the yellow matrix represents intergroup correlations between the same nodes. These red diagonals are collected and treated as similarities between nodes’ behaviors in different groups.

Our analysis involved a multistep process. We combined time series data from matching nodes of TBI (SLN or FMT) and control (sham) subjects to create a pseudo-subject to effectively calculate correlation matrices and assess connectivity changes for the same node cross-group (Fig. 1) (details in Supplementary Data S1).

To mitigate potential biases from the sequence of concatenation and account for variability in recovery status among the TBI-treated pigs, we introduced a shuffling mechanism with multiple trials and a drop-one-out strategy. Specifically, in each iteration, one subject was randomly excluded, and the remaining subjects were shuffled before recombination. This procedure was repeated 128 times, ensuring robustness against bias and variability from individual differences and outlier effects.

### Simulation

To underscore the efficacy of our cross-group temporal correlation analysis, we undertook a simulation study leveraging rs-fMRI data from 44 healthy piglets, matched in age to our study subjects and collected using identical rs-fMRI protocols.^19^ Using the same seven RSN nodes (detailed in Table 1), we generated time series for each node via the dual-regression procedure outlined earlier. These time series were normalized to have zero mean and a standard deviation of one.

To mimic disrupted functional activities indicative of TBI, we introduced Gaussian noise at varying intensities to the time series of three specific networks (executive control network; EXN, sensorimotor network; SMN, and default network; DMN). This created two simulated TBI groups to represent mild and severe injury levels, respectively. The entire dataset of 44 was then categorized into three as follows: a control (sham) group of 14 subjects without added noise, simulating normal brain function; a simulated mild TBI group of 14 subjects with low-level noise (standard deviation of 1); and a simulated severe TBI group of 16 subjects with high-level noise (standard deviations of 3, 5, 7, 10, and 15) to represent varying degrees of injury severity.

We conducted temporal correlation analyses between the sham group and each of the simulated TBI groups to validate our method’s statistical robustness in distinguishing between different levels of brain injuries.

### Spatial correlation analysis

In addition to the temporal correlation analysis using rs-fMRI, we performed a spatial correlation analysis by obtaining CBF maps associated with the seven RSNs using acquired ASL data. We masked the whole-brain CBF map for each subject with the seven RSNs. For each RSN, we used a similar methodology to the temporal correlation of rs-fMRI data for CBF spatial correlations (details in Supplementary Data S1).

## Results

**FIG. 2.**
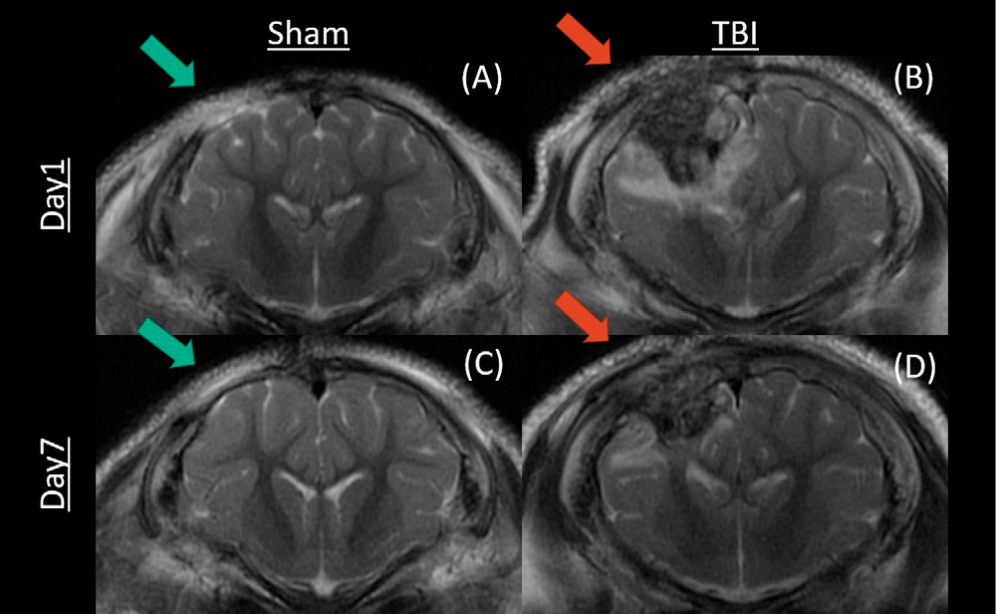
Representative visualizations of sham and traumatic brain injury (TBI) pigs. Left column: sham pig; right column: TBI pig; top row: day 1 images; bottom row: day 7 images. Blue arrows indicate the craniectomy surgical site, whereas red arrows point to the TBI injury. TBI, traumatic brain injury.

Pigs with TBI injuries showed significant brain lesioning and edema at D1 and D7 relative to sham pigs (Fig. 2). The pathophysiology of TBI pigs was consistent with moderate-to-severe TBI as previously reported by our research group.^16,17^

### Simulation assessment

**FIG. 3.**
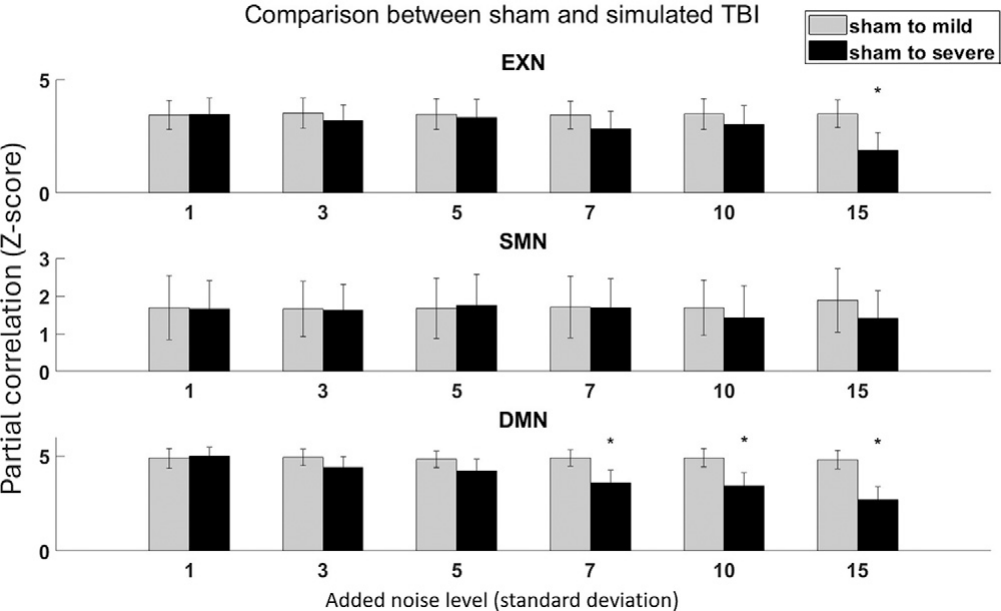
Simulation results of correlations between the sham to simulated mild and sham to simulated severe traumatic brain injury (TBI) injuries. Gray bars represent comparisons of sham to simulated mild TBI group. Black bars represent comparisons of sham to simulated severe TBI group at the added noise levels of standard deviations of 1, 3, 5, 7, 10, and 15 for each of the resting-state network (RSN) nodes. Generally, stable trends are observed for the gray bars, whereas descending trends are observed for the black bars in all three RSNs. These results indicate that similarities between the sham and TBI groups decreased with increasing simulated TBI severity. Star signs indicate statistically significant differences. RSN, resting-state network; TBI, traumatic brain injury.

The simulation results are displayed in Figure 3. The gray bars represent similarities between the simulated sham group and the simulated mild TBI group, whereas the black bars indicate similarities between the simulated sham group and the simulated severe TBI group at various noise levels (standard deviations of 1, 3, 5, 7, 10, and 15, respectively). The difference between the gray and the black bars consistently grows with the noise level in the severe TBI group across all three networks. This difference reaches statistical significance at noise level 15 for the EXN and 7 for the DMN in the severe TBI group.

### Temporal correlation analysis

**FIG. 4.**
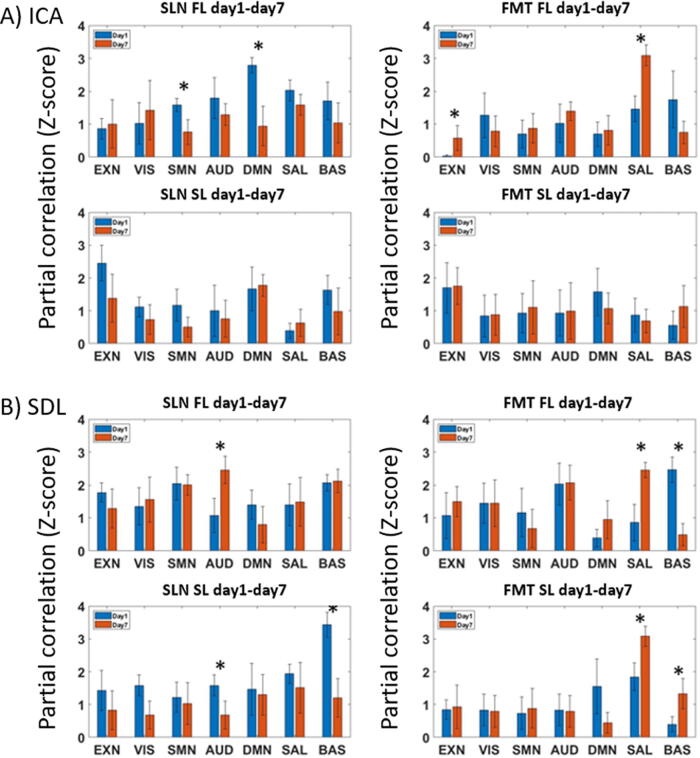
Temporal analysis results using (A) independent component analysis (ICA) and (B) sparse dictionary learning (sDL) approaches. In each section, the first row shows full dataset learning (FL) results, and the second row displays sham dataset learning (SL) results. The left side represents the TBI treated with saline (SAL) group, and the right side represents the TBI treated with fecal microbial transplant (FMT) group. Similarities between the sham group and the traumatic brain injury (TBI) group at day 1 are presented by blue bars and at day 7 by red bars. The *x*-axis shows the 7 RSN nodes, whereas the *y*-axis shows Fisher transformed partial correlations as Z-stats. Results are reported as means with error bars. Star signs indicate statistically significant differences with a *p*-value < 0.05. ICA, independent component analysis; RSN, resting-state network; sDL, sparse dictionary learning; TBI, traumatic brain injury.

The temporal correlation results for the TBI groups versus the sham group from D1 to D7, obtained by ICA and sDL analysis, are displayed in Figure 4A and B, respectively. The results represent the similarities between the sham group and the TBI group at D1 using blue bars and at D7 using red bars.

For the SLN-treated group, the SMN showed a significant decrease in the ICA result (Fig. 4A). Five out of seven RSNs displayed decreased correlations in both the FL and SL results, with SMN, AUD, and BAS being consistent. Meanwhile, no consistent increasing trends between FL and SL were identified in any other RSNs. In the sDL analysis of temporal analysis (Fig. 4B), the visual network (VIS), auditory network (AUD), and basal ganglia network (BAS) networks exhibited significant decreases in the SL results, whereas the remaining four RSNs showed a decreasing trend in the SL results.

For the FMT-treated group, the EXN and salience network (SAL) showed significant increases in FL results, whereas the networks EXN, SMN, and AUD had consistent increasing trends in both FL and SL results. No consistent decreasing trends between FL and SL were observed. In the sDL analysis, the SAL displayed a consistent, significant increase in both FL and SL results, while the EXN showed a consistently increasing trend between FL and SL results.

**FIG. 5.**
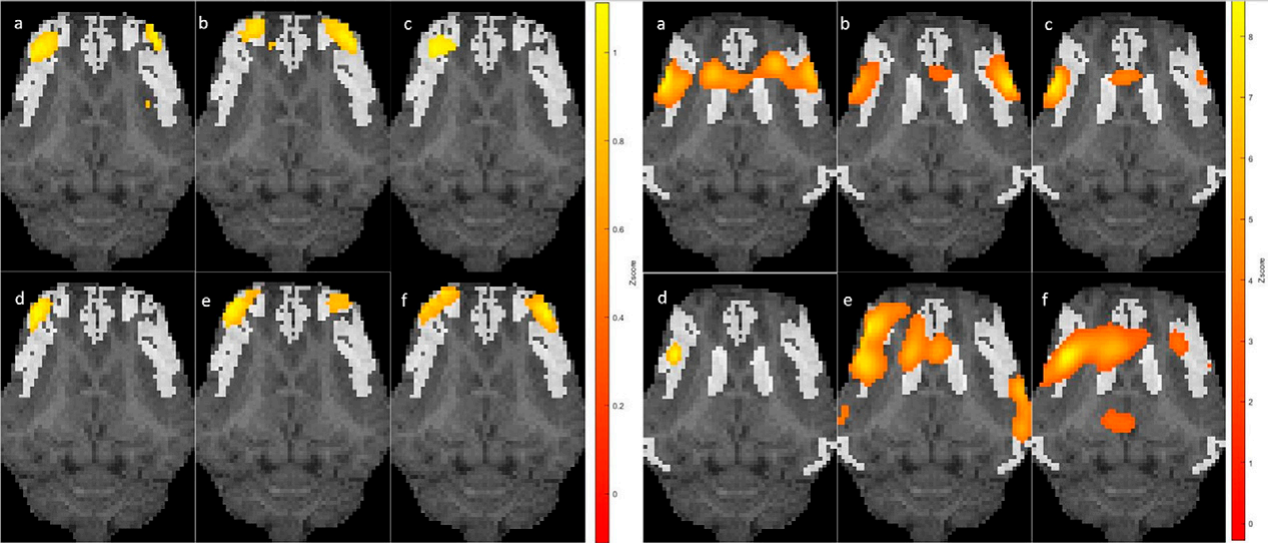
Representative activation maps for two resting-state networks (RSNs), EXN (executive control network, left) and SAL (salience network, right). Each subfigure displays **(a)** an traumatic brain injury (TBI) treated with saline (SLN) group pig, **(b)** a sham group pig, and **(c)** an TBI treated with fecal microbial transplant (FMT) group pig at day 1 (top row), **(d)** an SLN group pig, **(e)** a sham group pig, and **(f)** an FMT group pig at day 7 (bottom row). All correlation maps (color) are overlaid on a registered template pig anatomical image (gray). Sections overlaid on anatomical images are registered to an atlas for each RSN.

Overall, the temporal analysis indicates that the FMT group exhibits a consistent increase in the EXN, while the SMN and SAL show three instances of increases and one decrease. No network had more than two instances of decrease. All three increases of the SAL in the FMT group were statistically significant. For the SLN group, the SMN demonstrated consistent decreases across all four trials (ICA/sDL both with FL/SL), whereas the EXN, AUD, DMN, and BAS displayed three instances of decreases but one instance of increase. No network had more than two instances (half of the trials) of increases in the SLN group. The visualization of activation maps corresponding to two of the temporally changed nodes (EXN, SAL) is shown in Figure 5, providing an approximate impression of those nodes and associated changes.

### Spatial correlation analysis

**FIG. 6.**
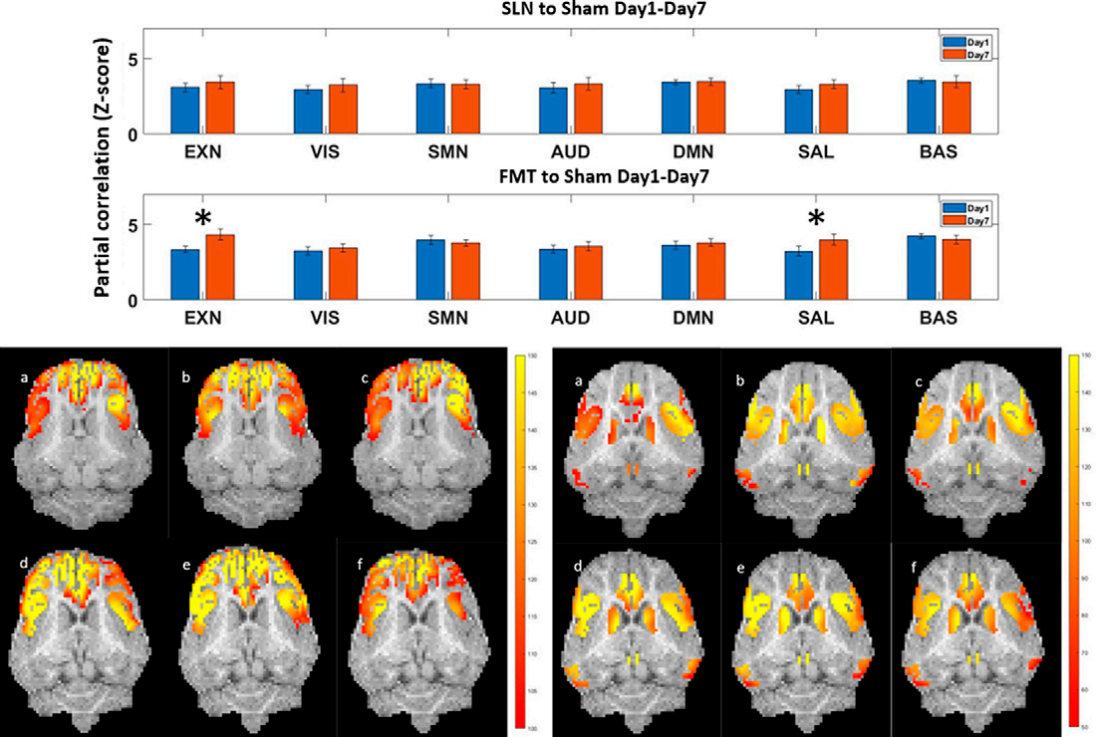
Spatial cerebral blood flow (CBF) correlation analysis for traumatic brain injury (TBI) treated with saline (SLN) (top bar plot) and TBI treated with fecal microbial transplant (FMT) (bottom bar plot) groups, and representative spatial CBF maps for two resting-state networks (RSNs), executive control network (EXN) (left) and salience network (SAL) (right). In the top graph, similarities between the sham group and the TBI groups at day 1 and day 7 are shown by the blue and red bars, respectively. Results are reported as means with error bars. Star signs indicate statistically significant differences with a *p*-value < 0.05. In the bottom visualization graph, each subfigure shows **(a)** an SLN group pig, **(b)** a sham group pig, and **(c)** an FMT group pig at day 1 (top row), **(d)** an SLN group pig, **(e)** a sham group pig, and **(f)** an FMT group pig at day 7 (bottom row). All CBF maps (color) are overlaid on a registered template pig anatomical image (gray).

The results of the spatial correlation analysis are presented in Figure 6. The top and bottom rows depict CBF spatial correlations for the seven RSNs in the SLN and FMT groups compared with the sham group, respectively. The lower part of Figure 5 presents visualized CBF maps for the EXN and SAL, providing an approximate impression of the spatial distribution and changes in these networks’ CBF correlations.

For the SLN group, no significant increases or decreases in CBF correlations were observed, suggesting that the SLN group without treatment did not lead to noticeable changes in CBF spatial correlations within the RSNs.

In contrast, the FMT group showed significant increases in correlation strength for the EXN and SAL from D1 to D7. This finding is consistent with the trends observed in the temporal correlation analysis (Fig. 4) for the same two RSNs, indicating that the FMT treatment may have a positive impact on the spatial correlation of CBF within these networks.

## Discussion and Conclusion

In our study, we assessed brain function recovery in a pig TBI model using temporal and spatial correlations. The findings indicate that FMT treatment resulted in enhanced recovery compared with the SLN group, as shown by increased functional connectivity correlations with the sham group from D1 to D7. Notably, consistent trends of improvement were observed in the EXN and SAL RSNs for the FMT group, with significant increases in both temporal and spatial correlations indicating potential recovery. Specifically, the SAL RSN showed significant temporal increases in three out of four trials and a notable spatial correlation increase, whereas the EXN RSN, despite not reaching statistical significance, displayed a consistent upward trend in temporal analysis and a significant spatial improvement.

Conversely, the SLN group exhibited consistent declines in functional indicators across the networks SMN, AUD, and DMN, suggesting worsening conditions post-TBI. These contrasting trends underscore the potential of FMT treatment in facilitating recovery and highlight the critical role of monitoring specific RSNs in evaluating TBI treatment efficacy.

Furthermore, the simulation results showed a consistent decrease in correlation (Fig. 3) with TBI progression as the noise intensity increases (in the simulation of TBI severity). Although the incorporation of Gaussian noise serves as a rudimentary approximation of TBI—given that actual injuries can introduce more complex noise patterns—our results remained consistent. This consistency indicates the effectiveness of the cross-group methodology in evaluating brain function recovery across all three RSNs. In addition, the similarity between the simulated mild group and the sham group also proved the stability of the cross-group temporal correlation methodology. These results demonstrate that the cross-group correlation study is a stable and reliable method for evaluating brain function recovery. The cross-group correlation could potentially have practical applications in clinical settings for assessing the effectiveness of TBI treatment strategies.

In this study, we identified the most relevant ICA components and SDL atoms based on their Pearson correlation with each of the seven RSNs and defined them as nodes (see Supplementary Fig. S1). The components/atoms were learned by group-level ICA and sDL approaches, with either the FL or the SL dataset on D1 and D7 together. Both ICA and sDL approaches decompose the group dataset into combinations of matrices (component matrix, atoms/dictionary matrix, and their corresponding coefficient matrix), yet they reveal distinct features because of their underlying principles. ICA tends to isolate more distinct and focused components, aligning with its goal to uncover independent signals within the data. Conversely, SDL often produces broader, more inclusive results, capturing larger activation areas that may encompass multiple features, thereby showing adaptability to smaller datasets. This contrast makes them complementary tools in our study and offers a multifaceted understanding of the recovery effect, allowing us to gain a comprehensive insight into brain function recovery post-TBI.

Using the sham group as a baseline for brain function comparison allowed the RSN nodes to represent normal brain functions, acknowledging that the sham group, reflecting the craniectomy’s effects, provides a more accurate baseline than a healthy control group. Nonetheless, the study faces limitations, notably the small size of the sham group (six subjects), limiting the effectiveness of deep learning approaches. In addition, the dual regression procedure’s reliance on the sham dataset for defining nodes might introduce errors because of anatomical variations between TBI and sham subjects, potentially leading to inaccurate time series and activation maps. Conversely, learning from the FL, which contains both sham and TBI groups, might highlight TBI-associated activities as fault “features” diverging from the objective of identifying nodes indicative of normal functions. This inclusion of TBI subjects introduces further uncertainty in distinguishing between normal and recovery-related brain functions, making it challenging to determine which dataset, SL or FL, more accurately represents recovery effects. Our findings did not definitively show that one dataset outperforms the other in characterizing brain function recovery. Acknowledging these challenges is crucial for interpreting the results and guiding future research. Future efforts should focus on increasing sample sizes, refining learning methods, or exploring new approaches to accurately represent normal brain functions.

It is noted that negative correlation coefficients existed in the raw correlation matrices obtained from temporal correlation analysis. Although positive correlation coefficients were considered to represent the similarity of the comparing components, negative correlation coefficients were ambiguous. Some studies^20^ suggest that these negative values represent a negative correlation in which one component increases as the other component decreases. Although some assumptions have been made in studies connecting the negative values with structural or functional connectivity in the brain,^21^ there are no straightforward built-in relationships between negative correlation effects and functional connectivity. We adopt the same methodology as previous studies by rejecting negative functional connectivity results to avoid uncertainty.^22,23^ However, it is essential to acknowledge the limitations of this approach and consider discussing the potential implications of negative correlation coefficients in future research. Exploring alternative methods for handling negative correlation coefficients or investigating whether they could provide additional insights into the functional connectivity of the brain may be valuable directions for future studies.

In this study, we reported increased correlations in only two out of a total of seven RSNs in pig brains post-TBI. However, because the evaluations were made only 6 days apart, it is possible that some RSNs may not have had adequate time to show significant changes. The high level of variability is possibly due to injury, and more robust changes may be seen with decreased variability in the later stage of recovering brains. Future studies may consider a longer period of recovery time for a thorough evaluation of functional activities and explore how these findings could be applied to other neurological disorders.

In summary, our study focused on evaluating post-TBI recovery, revealing the effectiveness of FMT treatment through the innovative use of temporal correlation analysis of rs-fMRI data alongside spatial correlation of CBF maps from ASL data. This temporal–spatial cross-group correlation approach not only confirmed the consistency of functional recoveries within RSNs across both simulated and experimental datasets but also underscored the utility of this novel method in accurately assessing functional activity changes following TBI and other neurological disorders. By offering a detailed insight into the recovery processes post-TBI, this method stands to significantly impact neuroscience, providing a robust framework for evaluating the outcomes of neural treatments.

## Transparency, Rigor, and Reproducibility Summary

The study was registered following the example of previous studies by Simchick, G., et al. (2021). “Detecting functional connectivity disruptions in a translational pediatric traumatic brain injury porcine model using resting-state and task-based fMRI.” Scientific reports 11 (1): 1–19.^1^ The analysis plan was likewise registered in accordance with this precedent.^2^ Eighteen (*n* = 18) pigs were involved in the study, and data were collected at two time points, that is, 1 and 7 days post-TBI.^6^ A simulation was conducted using an additional 44 pigs as well. All 18 pigs were included in the data analysis.^4^ No special equipment or software was used in process.^7^ The key inclusion criteria (e.g., primary diagnosis or prognostic factor) are established standards in field.^8^ Implications of possible violations of these assumptions include normal distribution of data and normal functional activities in the sham group^9^. Methods that do not require correction for multiple comparisons were used, including analysis of variance.^10^ At the time of writing, a replication study has not yet been planned.^11^ De-identified data from this study, along with the analytic code used to conduct the analyses, will soon be publicly available on GitHub.^12,13^ This article will be published under a Creative Commons Open Access license and, upon publication, will be freely available at https://www.liebertpub.com/loi/neu14

## Supplementary Material

Supplementary Data S1

## Supplementary Material

Supplementary Figure S1

## Abbreviations Used

ASL: Arterial spin labeling Acute care unit readmission
AUD: Auditory network
BAS: Basal ganglia networ
CBF: Cerebral blood flow
D1: Day 1
D7: Day 7
DMN: Default network
EXN: Executive control network
FL: Full dataset learning
FMT: TBI treated with fecal microbial transplant
ICA: Independent component analysis
rs-fMRI: Resting-state functional magnetic resonance imaging
RSNs: Restingstate networks
SAL: Salience network
sDL: Sparse dictionary learning
SL: Sham dataset learning
SLN: TBI treated with saline
SMN: Sensorimotor network
TBI: Traumatic brain injury
VIS: Visual network
