# Supplementary material for: Evaluation of Brain Function Recovery After Traumatic Brain Injury Treatment in a Porcine Model by Cross-Group Temporal–Spatial Correlation Analysis
Source: Neurotrauma Rep. 2024 Jul 1;5(1):617–27. doi: 10.1089/neur.2023.0059 (PMC11257111; doi:10.1089/neur.2023.0059)
Supplement: Supplementary Data S1 [file neur.2023.0059_sunsupplementarymaterials.docx]

**Supplemental materials**

**Temporal correlation analysis**

In our research, we first adopted a temporal correlation approach to examine the similarities in brain temporal activity between corresponding RSN nodes in the brains of the sham group and those undergoing TBI (SLN or FMT group). To accomplish this, we innovatively adapted the FSLNets tool, which was initially developed for comparing connectivity strength between different RSN nodes. Specifically, we modified FSLNets to assess the correlation of activity within the same RSN node across different individuals—one from a TBI group and another from the sham group—thereby creating a 'pseudo subject'. Generation of pseudo subjects allows FSLNets to identify correlations in node activity between the sham and TBI groups over time.

The process involves several steps: First, we paired time series from corresponding nodes of subjects in a TBI group with those in the sham group, concatenating these series to form a 2-dimensional matrix (2K by t) that simulates a single subject with twice the number of nodes (2K) for analysis by FSLNets. Second, using FSLNets’ functions, we calculated correlation matrices for these 2K nodes, which were then transformed into temporal Z-score matrices via Fisher’s transformation. A one-group T-test assessed the null hypothesis of no correlation, producing Z-stats that were interpreted within the context of our study’s adapted FSLNets pipeline. Next, from these matrices, we specifically focused on the Z-stats between corresponding nodes across the TBI and sham groups, shown as the diagonal red blocks at the lower-left quadrant of the correlation matrix (right section of Figure 1). These Z-stats, which traditionally gauge temporal functional similarity, were repurposed in our study to measure recovery or disruption in resting-state networks (RSNs) following TBI.

**Spatial correlation analysis**

The masked 3D CBF maps associated with each RSN were rearranged into 1D arrays (i.e., in the same format as time series) and then analyzed using the same FSLNets functions as in temporal analysis. Similar to the temporal correlation analysis, we aligned rearranged CBF data from the TBI group and the sham group to generate pseudo subjects, forming data matrices with dimensions 2K by v, where v is the number of voxels for the RSN in the ASL space. We then calculated inter-node correlation coefficient matrices for each RSN at both time points (D1 and D7). To reduce any potential bias in the cross-group correlation analysis, we conducted drop-one-out tests using 128 randomized group tests.


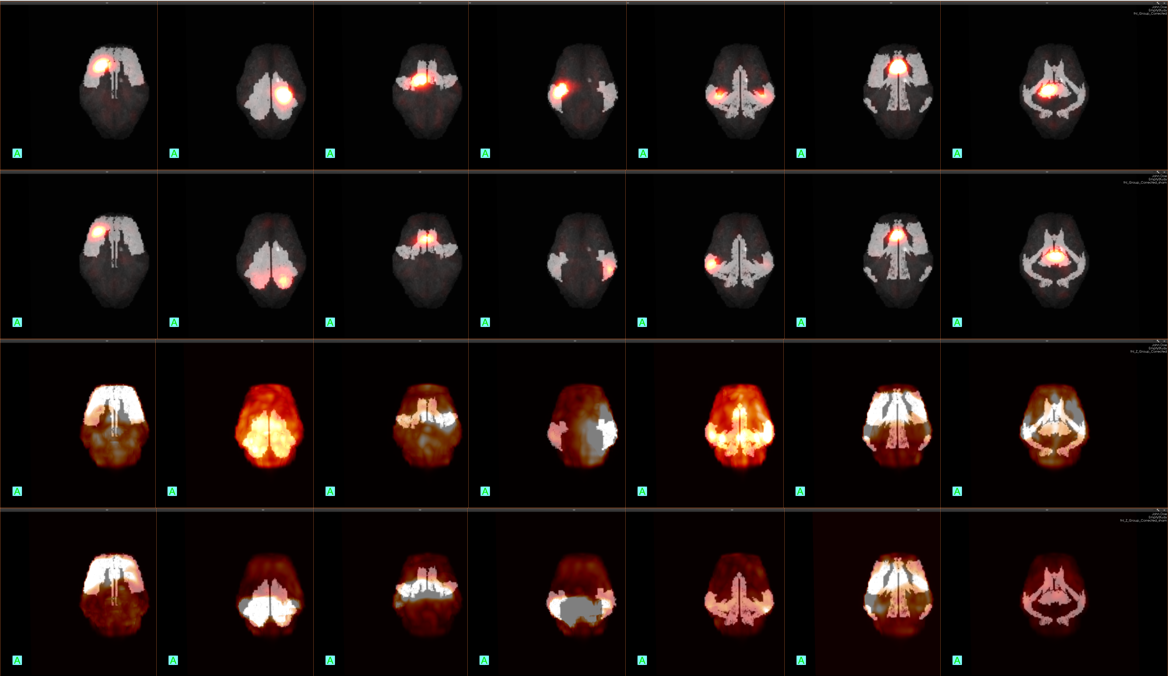


**Supplementary figure 1**. Visualization of overlapping each RSN’s atlas with the best correlated node from ICA and sDL group learning that was employed in the study. Columns from left to right represent RSNs: EXN, VIS, SMN, DMN, AUD, SAL, and BAS; rows from top to bottom: ICA with FL (full dataset learning), ICA with SL (sham-only dataset learning), sDL with FL, and sDL with SL. It is seen that the ICA nodes are more condensed and independent from each other, while the sDL nodes have larger distribution across the brain. The ICA nodes’ activation maps are usually only part of the RSN, while the sDL can fill most of the atlases. In ICA, no consistent clear difference can be observed between the FL and SL results. In sDL, SL shows more symmetric results in SMN and AUD, while other RSNs remain at a similar level in symmetry.
